# Integrated Assessment of Pollen Substitute Diets in Honey Bees Based on Transcriptional Responses, Immune-Derived Predicted Biological Age, and Survival

**DOI:** 10.3390/ani16193147

**Published:** 2026-10-08

**Authors:** Hyunjee Kim, Olga Frunze, Sang Mi Han, Soon Ok Woo, Jewon Jung, Hyung-Wook Kwon

**Affiliations:** 1 Department of Life Sciences & Convergence Research Center for Insect Vectors (CRCIV), Incheon National University R&D Complex, 265 Harmony-ro, Yeonsu-gu, Incheon 22014, Republic of Korea; beamed79@hanmail.net (H.K.); frunzeo1@inu.ac.kr (O.F.); 2 Apiculture Division, Department of Agricultural Biology, National Institute of Agricultural Sciences, Rural Development Administration (RDA), Wanju 55365, Republic of Korea; sangmih@korea.kr (S.M.H.); wooso1@korea.kr (S.O.W.); 3 Department of SmartBio, College of Life and Health Science, Kyungsung University, Busan 48434, Republic of Korea; jjewony@gmail.com

**Keywords:** acute heat stress, *Apis mellifera*, gene expression, honey bee nutrition, immune-derived predicted biological age, pollen substitute diet, survival

## Abstract

Heat events and nutritional stress threaten honey bee health, but how pollen substitute formulation shapes responses to acute heat remains unclear. Newly emerged *Apis mellifera* workers received three pollen substitute diets or two beebread-based reference diets for five days before exposure to 45 °C for 4 h. Expression of 12 genes related to nutrition, endocrine regulation, immunity, oxidative defense, and cellular stress was examined using mixed-effects and multivariate analyses. Immune-derived predicted biological age was estimated before and after heat exposure. Apparent consumption and survival were evaluated in an independent 18-day feeding assay without heat. Significant diet × heat interactions occurred for 10 genes, whereas *tor* and *dome* showed no significant interactions. At baseline, Diet2(M) exhibited a transcriptional profile similar to the control and a lower predicted biological age than Diet1(S) and Diet3(O). Diet2(M) also supported higher survival than Diet1(S), despite similar apparent consumption among substitute diets. These findings indicate that pollen substitute composition, rather than apparent consumption alone, can shape physiology and survival. Diet2(M) is therefore a priority candidate for colony-level evaluation, although improved heat tolerance was not demonstrated. These findings may guide the development of supplemental feeds supporting honey bee health under climate-related pollen scarcity and heat risk.

## Simple Summary

Extreme heat and limited pollen availability can threaten honey bee health, creating a need for nutritious diets that can supplement or replace natural pollen. We compared three experimental pollen substitute diets with two reference diets containing beebread, the naturally fermented pollen stored by honey bees. We examined changes in the activity of genes involved in nutrition, immunity, antioxidant defense, and heat responses before and after short-term heat exposure. We also measured diet consumption, estimated immune-derived biological age based on immune-related gene activity, and evaluated survival in a separate feeding experiment without heat exposure. Immune-derived biological age differed among diets, with Diet1(S) showing the highest value under baseline conditions, whereas Diet2(M) showed a relatively lower value. Among the experimental diets, Diet2(M), which contained defatted soybean fermented with *Bacillus subtilis* MBLB2796, showed a favorable combination of molecular characteristics and survival outcomes, including higher survival than Diet1(S). However, Diet2(M) did not demonstrate greater heat tolerance. These findings suggest that integrating molecular responses, immune-derived biological age, and survival may provide a useful approach for preliminary screening of pollen substitute diets. Further studies using whole colonies under field conditions are needed to confirm their practical benefits.

## 1. Introduction

Honey bees (*Apis mellifera* L.) provide essential pollination services that support agricultural production, biodiversity, and ecosystem stability [1]. Their health is nevertheless affected by interacting environmental pressures, including habitat degradation, pesticides, pathogens, nutritional deficiencies, and temperature extremes [2,3]. Individual worker bees generate metabolic heat through contractions of their thoracic flight muscles, while the coordinated activities of workers, including heat production, clustering, fanning, and evaporative cooling, contribute to colony-level thermoregulation [4,5]. Among these pressures, extreme heat is particularly relevant because it can challenge the collective thermoregulatory mechanisms that honey bee colonies use to maintain brood-nest temperature and increase the energetic and physiological demands placed on individual workers [4,5]. Under cage conditions, however, colony-level thermoregulatory behaviors are restricted; thus, the experiment primarily reflects the physiological responses of individual workers and small groups rather than the full thermoregulatory capacity of an intact colony. Nutritional condition may modify these responses because diet quality influences energy availability, antioxidant defense, immune function, and survival under elevated temperatures [6,7]. Supplemental feeding may therefore help support honey bee physiological function during periods when floral resources are limited and thermal stress is high.

Pollen stored within the hive undergoes microbial transformation to form beebread, an important source of nutrients for brood development and the physiological maintenance of adult honey bees [8,9]. This natural transformation suggests that microbial fermentation may offer a useful strategy for improving the nutritional properties of protein ingredients used in supplemental bee diets. Meanwhile, agricultural intensification can reduce the diversity and temporal continuity of pollen resources. Large-scale monocultures may expose colonies to pollen monodiets during short mass-flowering periods and subsequent forage scarcity, whereas the cultivation of non-forage crops, loss of flowering habitats, and pesticide use may further restrict the availability and accessibility of pollen. These effects may be compounded by climate-related changes in flowering phenology [10]. Beekeepers therefore use pollen substitutes to support colonies during periods of pollen scarcity [11,12]. The nutritional performance of these diets depends not only on consumption but also on ingredient composition, nutrient availability, digestibility, and post-ingestive utilization. Because apparent consumption reflects diet removal rather than nutrient assimilation, it cannot, by itself, establish nutritional efficacy [13,14]. A more informative assessment should therefore combine consumption data with physiological and longer-term performance indicators.

Dietary quality affects nutrient sensing, endocrine regulation, antioxidant defense, and innate immunity in honey bees [15,16,17]. Accordingly, the present study examined genes representing nutrient-sensing and endocrine pathways (*ilp1*, *ilp2*, *tor*, *jhamt*, *vg*, and *AmGr10*) and immune, oxidative-stress, and cellular stress responses (*relish*, *dome*, *defensin2*, *apid1*, *sod1*, and *hsp70*). These genes collectively represent physiological processes involved in nutrient allocation, behavioral maturation, immunity, and cellular stress responses [18,19,20,21]. However, transcriptional responses alone do not necessarily predict organismal performance [22]. Gene expression-based modeling has previously shown that molecular markers can be used to estimate maturation and biological age in honey bees [23]. Building on this approach, immune-derived predicted biological age provides a complementary, model-based estimate of physiological condition by integrating the expression of *relish*, *sod1*, *apid1*, and *dome* [24]. When considered together with consumption and survival, this index may serve as an early screening tool for evaluating pollen substitute performance. However, its utility in honey bee diet evaluation has not been established.

Our previous study, which was based on the same cage-feeding and acute heat-exposure experiment used in the present study, compared carbohydrate-only and protein-containing diets and characterized broad diet-dependent transcriptional responses for 11 genes under baseline and acute heat-stress conditions [25]. However, formal Diet × Heat interactions and their relationships with broader indicators of diet performance were not evaluated. To address these gaps, the present study focused on five protein-containing treatments: three pollen substitute formulations and two beebread-based reference diets. The previously reported expression data from these five treatments were reanalyzed to formally evaluate Diet × Heat interactions, and *dome* expression was newly quantified from the stored biological material, extending the molecular assessment to 12 genes. Transcriptional responses were considered together with five-day apparent consumption, immune-derived predicted biological age, and survival in an independent feeding assay performed under non-heat conditions. Apparent consumption and immune-derived predicted biological age are reported here for the first time, whereas the survival assay was conducted separately using an independent cohort of workers.

We investigated whether pollen substitute formulation influenced baseline transcriptional profiles and modulated the magnitude and direction of gene expression responses to acute heat exposure. We further examined these transcriptional patterns in relation to complementary indicators of diet performance. By integrating transcriptional profiles, immune-derived predicted biological age, apparent consumption, and survival, this study aimed to identify a candidate pollen substitute formulation for further evaluation and to assess the potential utility of predicted biological age as an early screening indicator. This integrated approach also distinguished acute transcriptional responses to heat exposure from broader indicators of nutritional performance.

## 2. Materials and Methods

### 2.1. Diet Preparation

Three pollen substitute diets—Diet1(S), Diet2(M), and Diet3(O)—were prepared using a common basal formulation and differed only in the soybean-derived ingredient, which accounted for 2% of each final diet (Table 1). The basal formulation comprised yeast-derived protein, a carbohydrate blend, lipid sources, liquid ingredients, functional additives, and a micronutrient mixture. The complete ingredient proportions and preparation procedures have been reported previously [25]. The analyzed crude protein contents were 17.77%, 17.76%, and 17.77% for Diet1(S), Diet2(M), and Diet3(O), respectively (Table S1). The three pollen substitute diets also had broadly comparable proximate and mineral compositions, supporting the soybean-derived ingredient as their principal formulation difference.

Diet1(S) contained Soytide powder (CJ Global Food & Bio, Seoul, Republic of Korea), a commercially produced soybean-derived product fermented with *Bacillus* spp. Diet2(M) contained defatted soybean powder fermented with *Bacillus subtilis* MBLB2796, a honey bee gut-derived strain previously evaluated for its potential application as a functional feed microorganism [26]. The fermented material was freeze-dried before incorporation into the diet. Diet3(O) contained non-fermented defatted soybean powder [27]. The formulations used in the present study were identical to those reported previously [25].

Beebread alone (Bbread) and beebread provided together with 30% sugar syrup (Cont) were included as reference treatments. The Bbread treatment consisted solely of beebread collected from three clinically healthy colonies maintained at the experimental apiary. In the Cont treatment, beebread and 30% sugar syrup were offered ad libitum in separate feeders, providing simultaneous access to pollen-derived nutrients and a supplemental carbohydrate source. All dietary materials were stored in airtight containers at 4 °C until use.

### 2.2. Cage Feeding, Acute Heat Exposure, and Sample Collection

The cage-feeding and acute heat-exposure procedures and the associated biological samples were derived from the same experiment reported previously [25]. The present study focused on the five protein-containing treatments from that experiment for additional and integrative analyses. One capped-brood frame was collected from each of three clinically healthy western honey bee (*Apis mellifera*) colonies maintained at the Incheon National University Apiary, Republic of Korea, and transferred to an incubator for adult emergence. Each source colony was headed by a queen reared in the previous year and in its second season at the time of the experiment. The precise subspecific or genetic background of the colonies was not determined. We maintained all colonies under the same standard apiary management conditions, and we applied no experimental dietary supplementation, medication, or other specific treatment before brood-frame collection. The colonies showed normal brood patterns and no visible clinical signs of disease at the time of collection. Workers that emerged within a 24 h period from the three source colonies were pooled and thoroughly mixed before being randomly allocated to plastic cages (17 cm × 14.5 cm × 9.5 cm), with 30 workers per cage. Three cages were assigned to each dietary treatment, and each cage served as a biological replicate. Honey bees were maintained in darkness at 33 ± 2 °C and 55 ± 5% relative humidity. This temperature was selected to provide standardized conditions during early adult development and to maintain consistency with the cage-feeding protocol used in our previous study [25].

Each cage received approximately 20 g of the assigned diet, provided ad libitum for five days. The five-day feeding period was selected to provide standardized dietary exposure during early adult development and was consistent with the cage-feeding protocol used in our previous study [25]. Water was continuously supplied through an inverted 15 mL tube fitted with a perforated cap. Diet containers were weighed before placement in the cages and again at the end of the feeding period. The difference between the initial and final weights was recorded as cumulative apparent consumption per cage over the five-day feeding period. Apparent consumption was not normalized to bee-days or adjusted for mortality. Because evaporation and incidental spillage could not be quantified separately, this measure reflects diet disappearance rather than actual ingestion.

**Figure 1:**
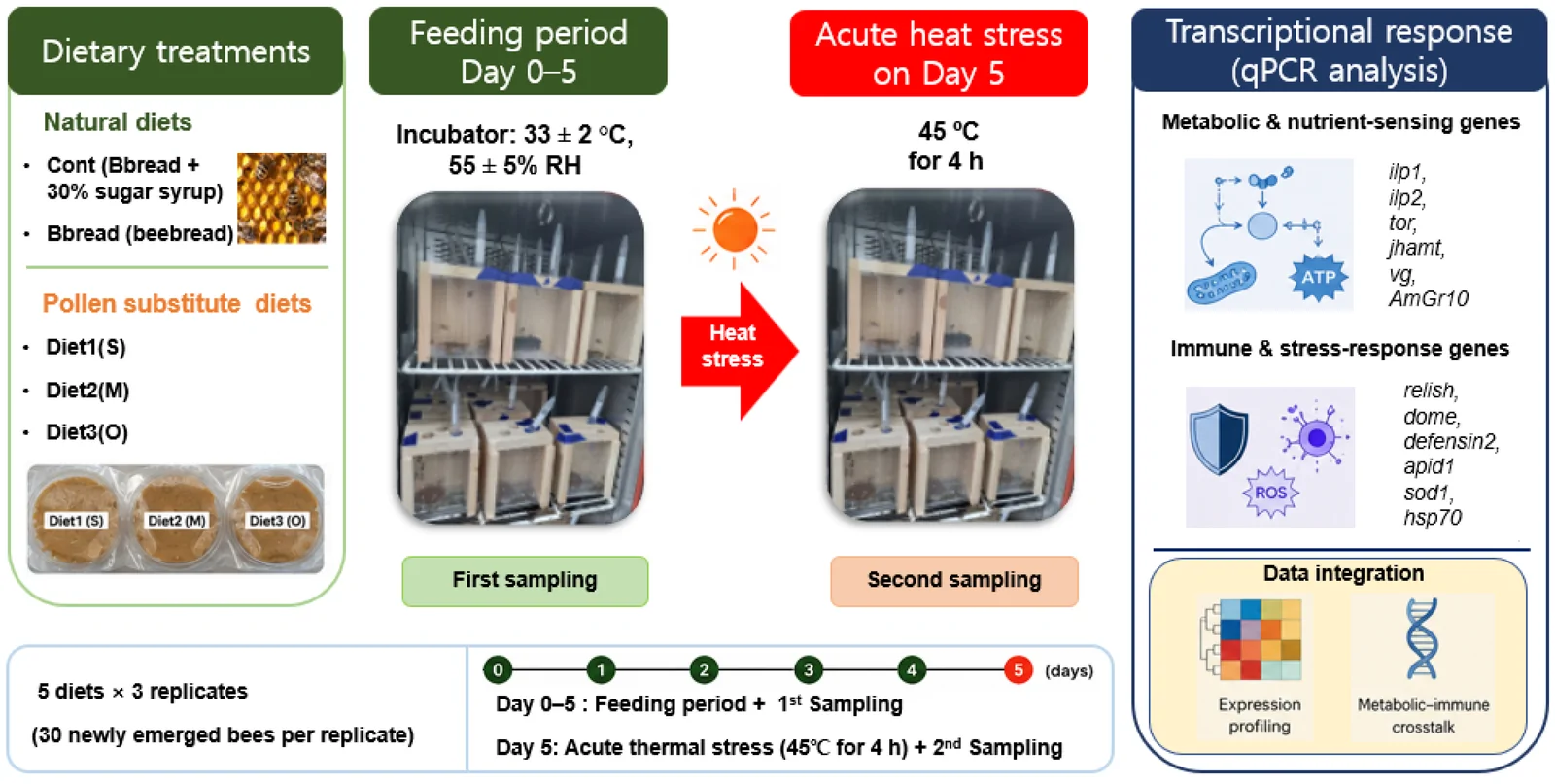
Overview of the feeding, heat-exposure, and survival experiments. Newly emerged workers were fed one of five diets for five days and sampled before and after exposure to 45 °C for 4 h. Each diet was represented by three cages. Survival was assessed separately over 18 days in honey bees that were not exposed to heat.

Before heat exposure on Day 5, ten workers were collected from each cage to represent the baseline condition. The remaining workers were exposed to 45 °C for 4 h following the acute heat-stress procedure described by Alqarni et al. [28]. Mortality was assessed immediately at the end of the 4 h heat exposure, before post-exposure sampling. Immediately after exposure, an additional ten workers were collected from each cage. Samples from both thermal conditions were immediately immersed in liquid nitrogen and stored at −80 °C until molecular processing. The overall experimental design is presented in Figure 1.

### 2.3. Tissue Preparation, RNA Isolation, and cDNA Synthesis

At each sampling point, ten workers were randomly collected from each cage, of which five were used for gene expression analysis. Each cage served as an independent biological replicate and contributed paired baseline and post-heat samples. Thus, thermal condition was treated as a within-cage repeated factor. Three replicate cages were included per dietary treatment (15 cages in total), and each cage was sampled twice, yielding *n* = 3 paired cage-level observations in each of the 10 diet × thermal-condition cells (30 cage-condition observations per gene). The collected honey bees were immediately transferred to 15 mL conical tubes, immersed in liquid nitrogen, and stored at −80 °C until tissue preparation. Samples from individual cages and thermal conditions were processed separately throughout the experiment.

For head-specific analysis, three frozen workers from each cage were decapitated on dry ice, and their heads were pooled to generate one head sample. The remaining two intact workers were pooled separately to generate one whole-body sample. Head samples were used to analyze *jhamt*, *tor*, *ilp1*, *ilp2*, *hsp70*, and *AmGr10*, whereas whole-body samples were used to analyze *vg*, *relish*, *defensin2*, *apid1*, *sod1*, and *dome*. Expression data for the 11 target genes other than *dome* were reported previously [25], whereas *dome* expression was newly quantified from the stored cDNA samples in the present study. Total RNA was isolated from each pooled sample using the RNeasy Mini Kit (Qiagen, Valencia, CA, USA) according to the manufacturer’s instructions. RNA concentration and purity were determined spectrophotometrically, and samples with A260/A280 ratios between 1.8 and 2.0 were used for cDNA synthesis. Complementary DNA was synthesized from 1 μg of total RNA using the RNA to cDNA EcoDry™ Premix (Oligo dT) kit (Takara Bio USA, Inc., San Jose, CA, USA) in a final reaction volume of 20 μL, following the manufacturer’s protocol. The resulting cDNA samples were stored at −20 °C until qPCR analysis. Expression of dome was subsequently quantified using whole-body cDNA corresponding to the same cage-level biological replicates.

### 2.4. Quantitative Real-Time PCR

The expression levels of the 12 target genes were quantified by quantitative real-time PCR (qPCR). Each reaction was performed in a total volume of 20 μL containing 2 μL of cDNA template, 10 μL of Brilliant III Ultra-Fast SYBR Green QPCR Master Mix (Agilent Technologies, Cedar Creek, TX, USA), 10 pmol each of the gene-specific forward and reverse primers, and nuclease-free water. Amplification was conducted using an AriaMx Real-Time PCR System (Agilent Technologies, Santa Clara, CA, USA), with an initial denaturation at 95 °C for 5 min, followed by 40 cycles of 95 °C for 30 s, 60 °C for 30 s, and 72 °C for 30 s. A melting-curve analysis was performed after amplification to confirm product specificity.

Relative expression was normalized to *β-actin*, consistent with our previously reported experimental protocol [25], and calculated using the 2^−ΔΔCt^ method. However, the stability of *β*-actin across the dietary and thermal conditions was not independently validated before normalization. Each cage-level biological sample was analyzed in three technical replicates, and the mean Ct value was used for subsequent analyses. Primer sequences and their sources are provided in Table S2.

### 2.5. Estimation of Immune-Derived Predicted Biological Age

For each cage-level biological replicate, immune-derived predicted biological age was estimated from the mean Ct values of *dome*, *relish*, *sod1*, and *apid1*. The Ct values were normalized using the reference gene *β-actin* and processed according to the published procedure before application of the trained elastic net regression model developed by Frunze et al. [24]. The published preprocessing procedure and model coefficients were applied without modification. Separate predictions were generated for each cage under baseline and post-heat conditions. These predictions were interpreted as model-derived indicators of immune-associated physiological condition rather than direct measures of chronological age.

### 2.6. Survival Assay

An independent survival assay was conducted at a separate time using a newly collected cohort of workers, with the same dietary treatments and cage-feeding conditions described above. Thirty newly emerged workers were assigned to each cage, with three cages per dietary treatment. Honey bees were maintained on their assigned diets without heat exposure, and survival was recorded daily for 18 days.

### 2.7. Statistical Analysis

Statistical analyses were performed using XLSTAT (v2023.2.0, Addinsoft, Paris, France, 2023). For each gene, diet × heat effects on ΔCt values were evaluated using a linear mixed-effects model with diet, thermal condition, and their interaction as fixed effects and cage identity as a random intercept. Thermal condition was treated as a within-cage repeated factor because paired baseline and post-heat samples were collected from the same cages, as described in Section 2.3 (*n* = 3 paired cage-level observations per diet × thermal-condition cell). Technical qPCR replicates were averaged before analysis. Interaction *p*-values were adjusted across the 12 genes using the Benjamini–Hochberg false discovery rate (FDR) procedure. Multiplicity-adjusted estimated marginal means were used for post hoc comparisons when appropriate. Relative expression was calculated using the baseline Cont treatment as the calibrator; log_2_-transformed values were used only for graphical presentation. Within each thermal condition, gene expression and immune-derived predicted biological age were compared among diets using one-way ANOVA followed by Tukey’s honestly significant difference (HSD) test. Model assumptions were assessed by visually inspecting residual distributions and residual-versus-fitted plots.

PCAmix was used to visualize the overall transcriptional separation between thermal conditions, followed by separate principal component analyses within each condition. Cage-level expression values were centered and scaled. Hierarchical clustering was performed using Euclidean distance and Ward’s linkage, and heatmaps were generated from gene-wise Z-score-standardized expression values. These multivariate analyses were descriptive.

Apparent consumption was compared among Bbread, Diet1(S), Diet2(M), and Diet3(O) using one-way ANOVA followed by Tukey’s HSD test; Cont was excluded because beebread and sucrose solution were provided separately. For each cage, restricted mean survival time (RMST) through day 18 was calculated as the area under the cage-specific survival curve and compared among diets using one-way ANOVA followed by Tukey’s HSD test. Kaplan–Meier curves were used for visualization. Honey bees alive on day 18 were right-censored in the Kaplan–Meier curves. Statistical significance was set at *p* < 0.05.

## 3. Results

### 3.1. Apparent Diet Consumption During the Five-Day Feeding Period

Apparent diet consumption differed significantly among Bbread and the three pollen substitute treatments during the five-day feeding period (F(3, 8) = 12.98, *p* = 0.003). Apparent consumption of Bbread was significantly greater than that of each pollen substitute diet, whereas Diet1(S), Diet2(M), and Diet3(O) did not differ significantly from one another (Tukey’s HSD test, *p* > 0.05; Table 2).

### 3.2. Diet-Dependent Transcriptional Profiles Under Baseline and Acute Heat-Stress Conditions

**Figure 2:**
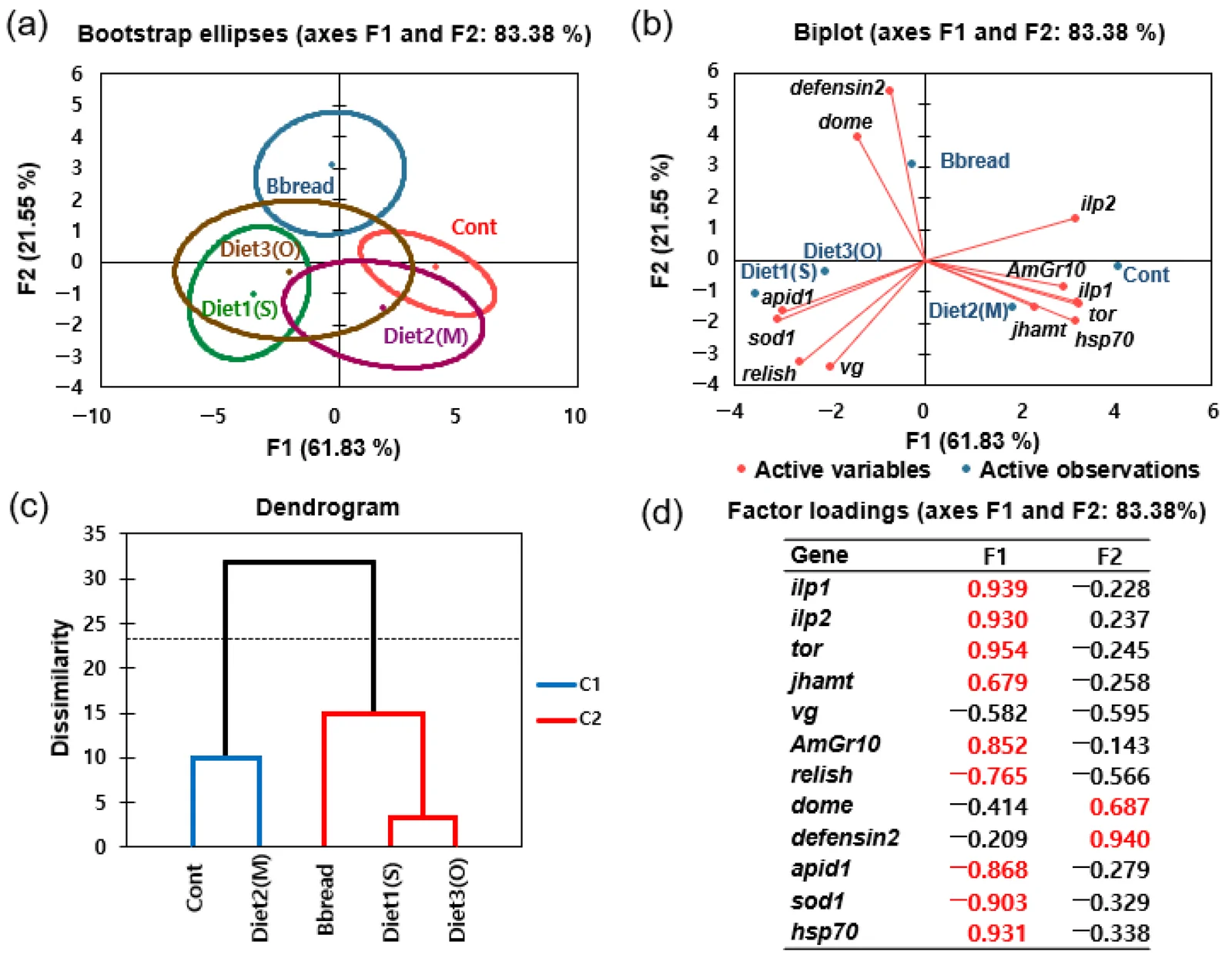
Multivariate transcriptional profiles after five days of dietary treatment under baseline conditions. Cage-level expression values for 12 genes were centered and scaled before analysis (*n* = 3 cages per diet). (**a**) PCA score plot with bootstrap ellipses. (**b**) Biplot showing dietary treatments and gene loadings. (**c**) Hierarchical clustering based on Euclidean distance and Ward’s linkage. (**d**) Gene loadings on F1 and F2. F1 (61.83%) and F2 (21.55%) together explained 83.38% of the total variance. Absolute loading values ≥0.60 are highlighted in red and were considered salient contributors to each principal component.

**Figure 3:**
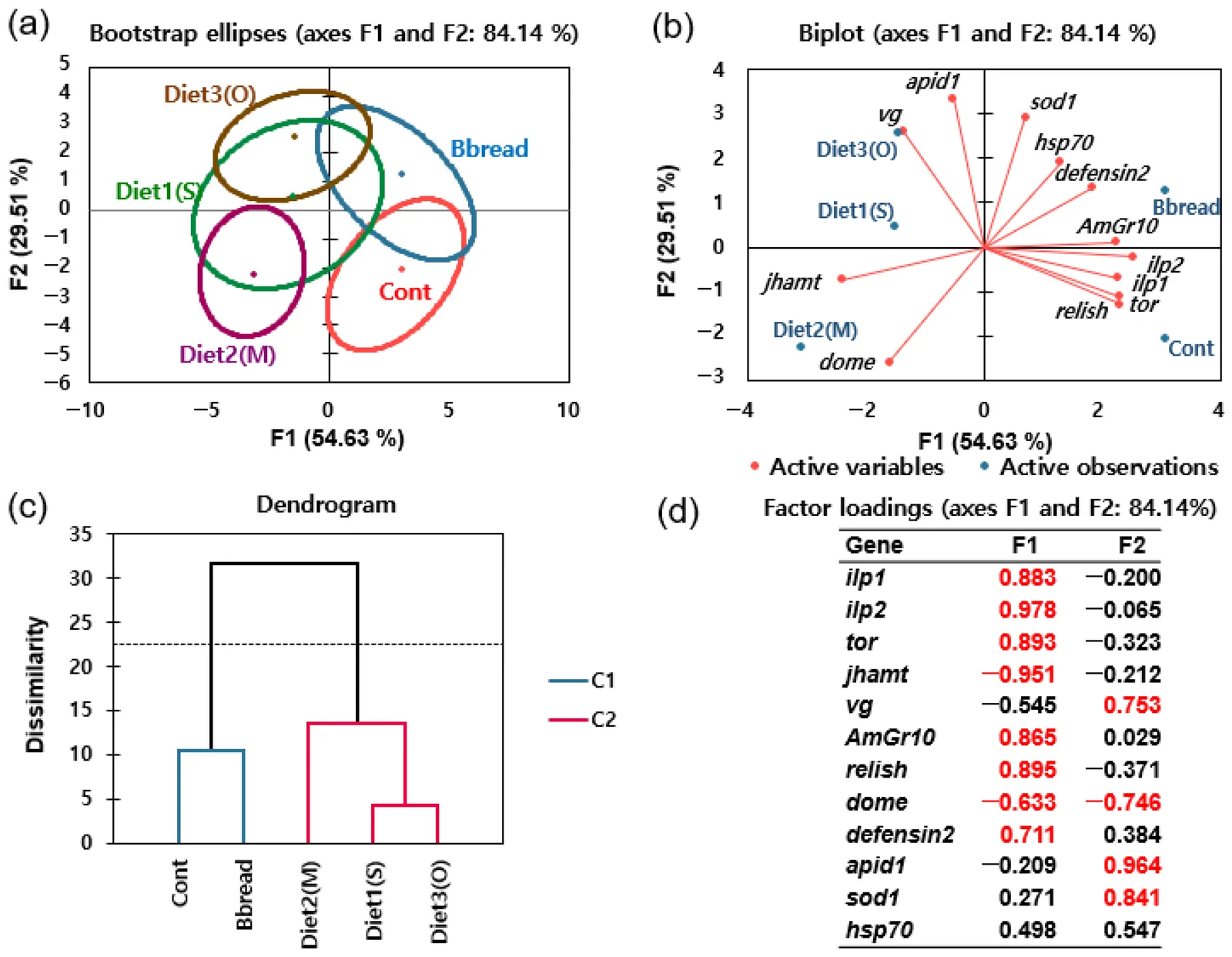
Multivariate transcriptional profiles following acute heat exposure. Cage-level expression values for 12 genes were centered and scaled before analysis (*n* = 3 cages per diet). (**a**) PCA score plot with bootstrap ellipses. (**b**) Biplot showing dietary treatments and gene loadings. (**c**) Hierarchical clustering based on Euclidean distance and Ward’s linkage. (**d**) Gene loadings on F1 and F2. F1 (54.63%) and F2 (29.51%) together explained 84.14% of the total variance. Loading values with an absolute magnitude of ≥0.60 are highlighted in red and were considered salient contributors to each principal component.

PCAmix analysis of the combined dataset separated the baseline and heat-stress conditions, with the first two dimensions explaining 68.11% of the total variation (Figure S1). Separate principal component analyses were then used to examine diet-dependent patterns within each condition (Figure 2 and Figure 3). Under baseline conditions, F1 and F2 explained 61.83% and 21.55% of the variation, respectively (83.38% cumulatively; Figure 2a,b). Cont and Diet2(M) were positioned on the positive side of F1, Diet1(S) and Diet3(O) predominantly on the negative side, and Bbread primarily on the positive side of F2. Hierarchical clustering similarly grouped Cont with Diet2(M), whereas Bbread, Diet1(S), and Diet3(O) formed the second major cluster (Figure 2c).

Using an absolute loading threshold of ≥0.60, baseline F1 was characterized by salient positive loadings for *tor* (0.954), *ilp1* (0.939), *hsp70* (0.931), *ilp2* (0.930), *AmGr10* (0.852), and *jhamt* (0.679), and salient negative loadings for *sod1* (−0.903), *apid1* (−0.868), and *relish* (−0.765) (Figure 2d). F2 was characterized primarily by positive loadings for *defensin2* (0.940) and *dome* (0.687). Accordingly, the positive region of F1 was associated mainly with nutrient-sensing, metabolic, endocrine, and cellular stress-response genes, whereas the negative region was associated with immune and oxidative-defense genes.

Following acute heat exposure, F1 and F2 explained 54.63% and 29.51% of the variation, respectively (84.14% cumulatively; Figure 3). Cont and Bbread were positioned on the positive side of F1, whereas Diet1(S) and Diet3(O) remained predominantly on the negative side. Diet2(M) occupied the lower-left region of the ordination, distinct from both reference treatments and the other pollen substitute diets. Hierarchical clustering grouped Cont with Bbread and placed the three pollen substitute diets in a separate major cluster (Figure 3c).

Using the same absolute loading threshold of ≥0.60, F1 showed salient positive loadings for *ilp2* (0.978), *relish* (0.895), *tor* (0.893), *ilp1* (0.883), *AmGr10* (0.865), and *defensin2* (0.711), and salient negative loadings for *jhamt* (−0.951) and *dome* (−0.633) (Figure 3d). F2 was characterized by salient positive loadings for *apid1* (0.964), *sod1* (0.841), and *vg* (0.753), and a salient negative loading for *dome* (−0.746).

Diet-dependent separation therefore persisted after heat exposure, although treatment positions and the genes contributing most strongly to the principal components changed between thermal conditions.

### 3.3. Hierarchical Clustering of Gene Expression Profiles Across Dietary Treatments

**Figure 4:**
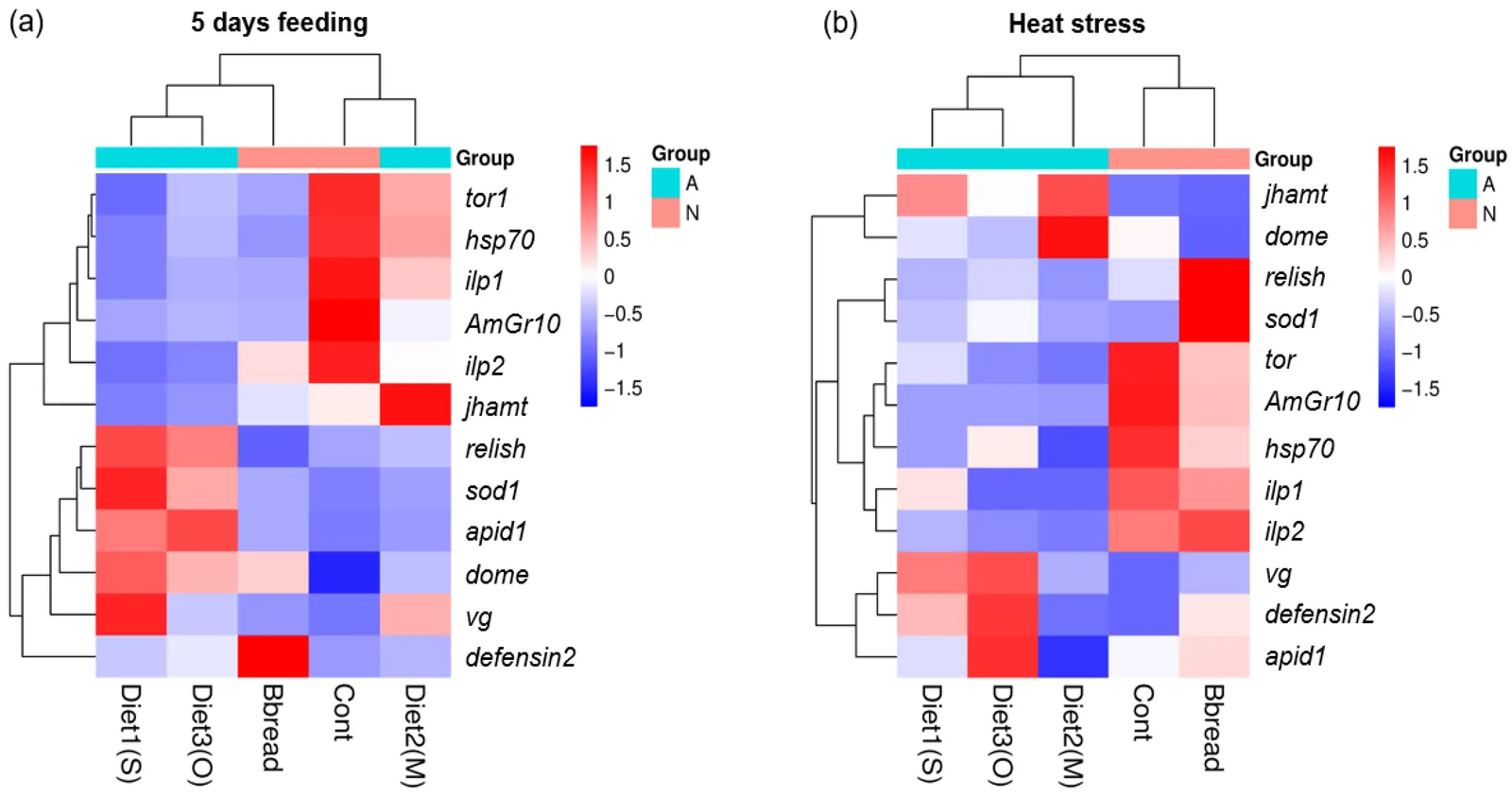
Hierarchical clustering heatmaps of gene-expression profiles under (**a**) baseline conditions after five days of feeding and (**b**) acute heat exposure. Rows represent genes and columns represent dietary treatments. Gene-wise Z-score-standardized expression values range from relatively low (blue) to high (red); dendrograms indicate similarity among genes and treatments. The top annotation bar identifies artificial pollen substitute diets (A: Diet1(S), Diet2(M), and Diet3(O)) and natural beebread-based reference treatments (N: Bbread and Cont).

Hierarchical clustering revealed condition-dependent relationships among dietary treatments (Figure 4). At baseline, Cont and Diet2(M) formed a closely related pair. Cont showed relatively high expression of *tor*, *hsp70*, *ilp1*, *AmGr10*, and *ilp2*, whereas Diet2(M) showed moderately similar patterns for several of these genes and comparatively high *jhamt* expression. Diet1(S) and Diet3(O) formed another pair, characterized by relatively high expression of *relish*, *sod1*, *apid1*, and *dome*; *vg* expression was highest in Diet1(S). Bbread joined this branch at a higher clustering level and was distinguished mainly by high *defensin2* expression.

Following acute heat exposure, Cont and Bbread formed one major cluster, although their expression patterns remained distinct (Figure 4b). The three pollen substitute diets formed the other major cluster. Diet1(S) and Diet3(O) grouped closely and showed relatively high *vg* and *defensin2* expression; Diet1(S) also showed elevated *jhamt* expression, whereas Diet3(O) showed particularly high *apid1* expression. Diet2(M) joined this pair at a higher clustering level and was characterized by relatively high *jhamt* and *dome* expression.

Thus, treatment relationships changed with thermal condition. Diet2(M) grouped with Cont at baseline, whereas the three pollen substitute diets formed one major cluster after heat exposure. Diet1(S) and Diet3(O) remained closely associated under both conditions, broadly consistent with the PCA results.

### 3.4. Diet × Heat-Stress Interactions in Metabolic, Immune, and Stress-Response Gene Expression

**Figure 5:**
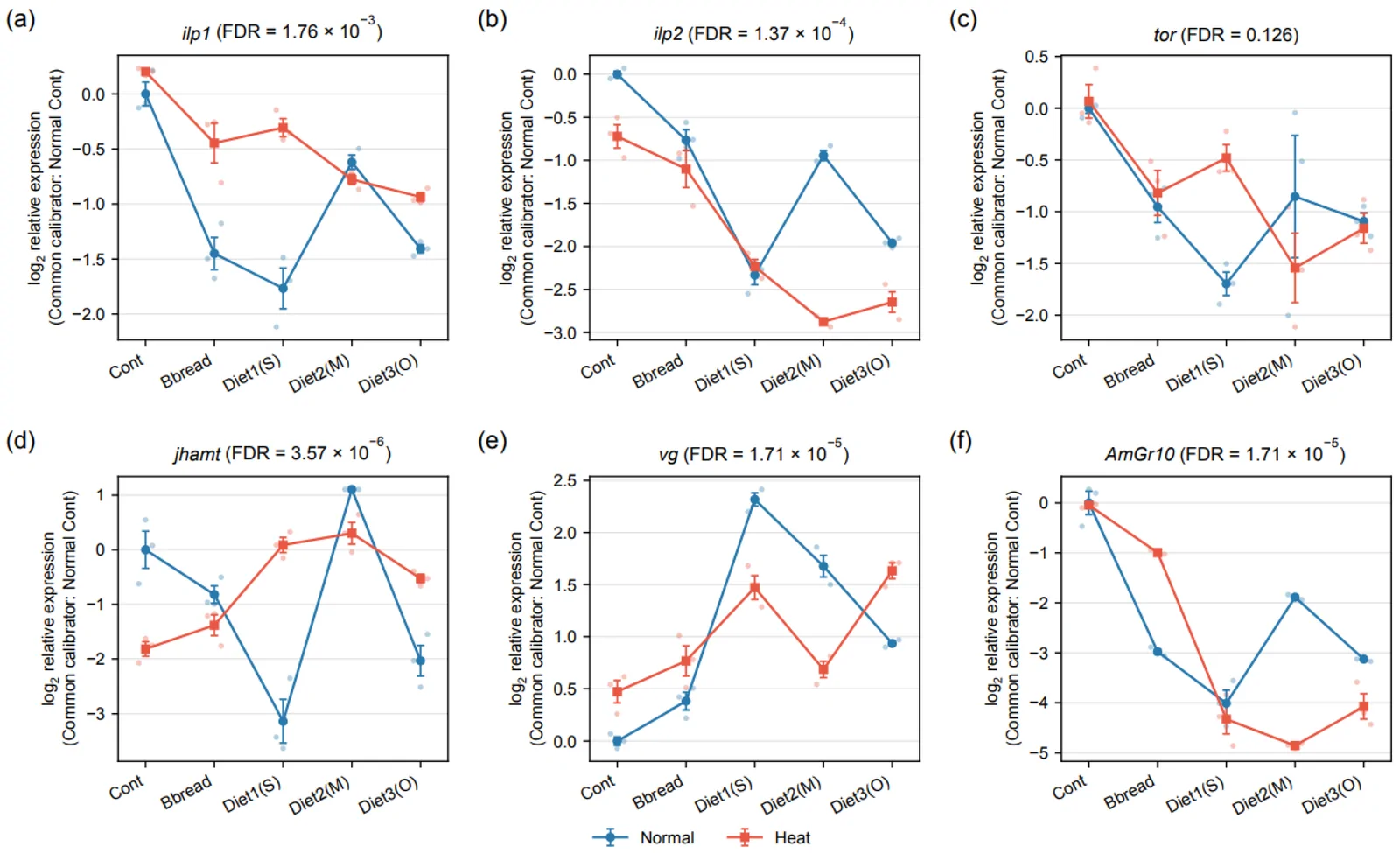
Diet × heat stress interactions in the expression of metabolic and nutrient-sensing genes. Expression profiles of (**a**) *ilp1*, (**b**) *ilp2*, (**c**) *tor*, (**d**) *jhamt*, (**e**) *vg*, and (**f**) *AmGr10* under baseline conditions (blue circles) and following acute heat exposure (red squares). Large symbols represent group means, error bars represent the standard errors of the means, and small points represent individual biological replicates (*n* = 3 cages per diet). Expression values are presented on a log_2_ scale relative to the baseline Cont group. The FDR-adjusted *p*-value in each panel corresponds to the diet × heat interaction tested using ΔCt values, with Benjamini–Hochberg adjustment across the 12 genes.

**Figure 6:**
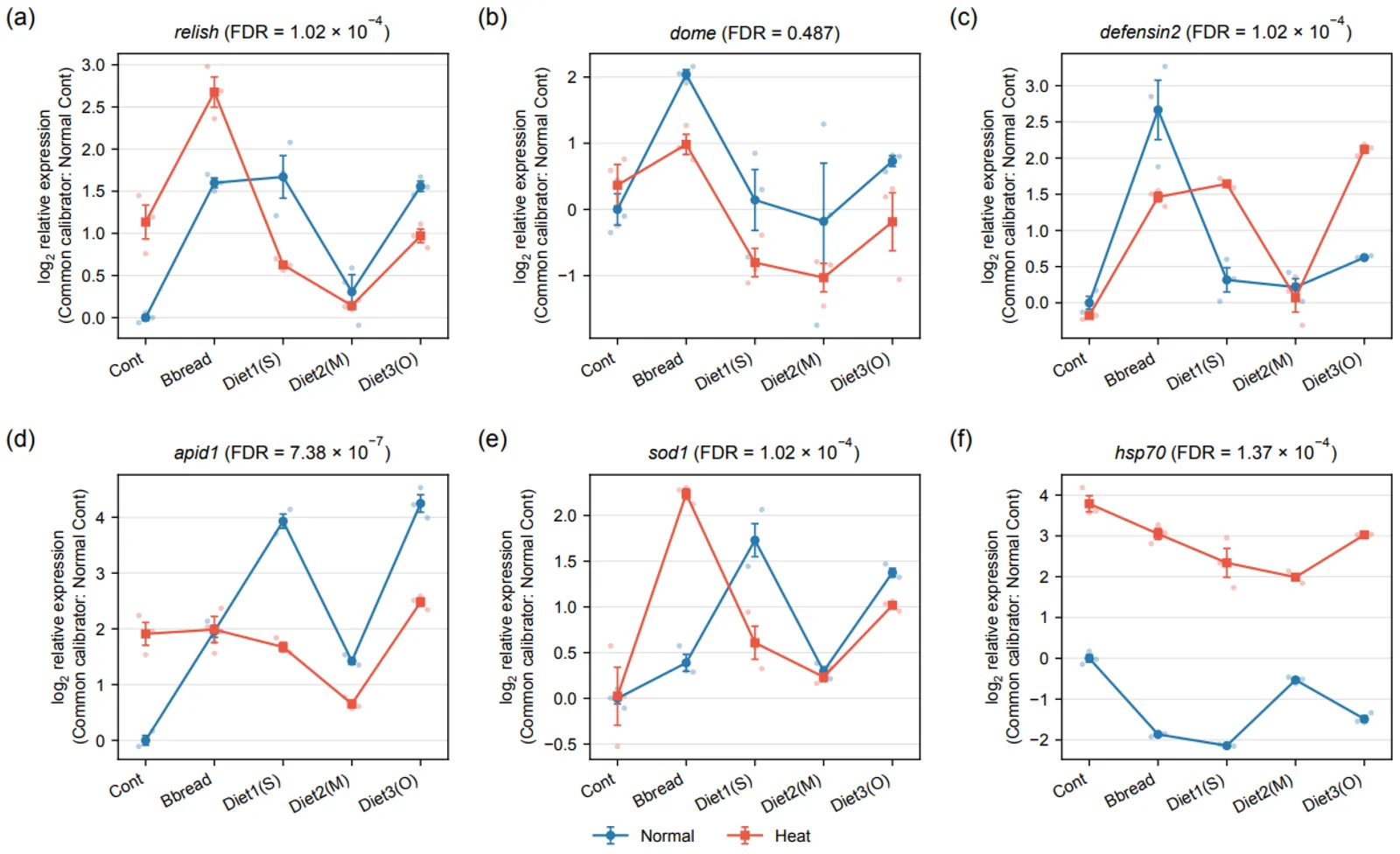
Diet × heat stress interactions in the expression of immune and stress-response genes. Expression profiles of (**a**) *relish*, (**b**) *dome*, (**c**) *defensin2*, (**d**) *apid1*, (**e**) *sod1*, and (**f**) *hsp70* under baseline conditions (blue circles) and following acute heat exposure (red squares). Large symbols represent group means, error bars represent the standard errors of the means, and small points represent individual biological replicates (*n* = 3 cages per diet). Expression values are presented on a log_2_ scale relative to the baseline Cont group. The FDR-adjusted *p*-value in each panel corresponds to the diet × heat interaction tested using ΔCt values, with Benjamini–Hochberg adjustment across the 12 genes.

No mortality occurred in any dietary treatment during the 4 h heat exposure. Significant diet × heat interactions were detected for 10 of the 12 genes and remained significant after FDR correction (Figure 5 and Figure 6; Table S3). The interactions for *tor* (FDR-adjusted *p* = 0.126) and *dome* (FDR-adjusted *p* = 0.487) were not significant. Among the significant interactions, *apid1* had the smallest FDR-adjusted *p*-value (7.38 × 10^−7^), indicating that heat-associated transcriptional responses differed among diets for most genes.

Among the metabolic and nutrient-sensing genes (Figure 5), mean *ilp1* expression was higher following heat exposure in Cont, Bbread, Diet1(S), and Diet3(O), but slightly lower in Diet2(M). Mean *ilp2* expression was generally lower following heat exposure, with the largest differences observed in Diet2(M) and Diet3(O). The mean heat-associated difference in *tor* expression was relatively small, and its diet × heat interaction was not significant after FDR correction. Mean *jhamt* expression was higher following heat exposure in Diet1(S) and Diet3(O) but lower in the other treatments. Mean *vg* expression was higher in Cont, Bbread, and Diet3(O) but lower in Diet1(S) and Diet2(M), whereas mean *AmGr10* expression was markedly higher in Bbread but lower in all three pollen substitute groups.

Among the immune and stress-response genes (Figure 6), mean *relish* expression was higher following heat exposure in Cont and Bbread but lower in the pollen substitute groups. Mean *dome* expression was higher following heat exposure across all diets; however, the nonsignificant interaction indicated insufficient evidence that the magnitude of this response differed among diets. Mean *defensin2* expression was higher following heat exposure in Diet1(S) and Diet3(O), lower in Bbread, and showed relatively small differences in Cont and Diet2(M).

Mean *apid1* expression was higher following heat exposure in Cont, showed a relatively small difference in Bbread, and was lower in Diet1(S), Diet2(M), and Diet3(O). Mean *sod1* expression was markedly higher in Bbread, lower in Diet1(S) and Diet3(O), and showed a relatively small difference in Diet2(M). Mean *hsp70* expression was higher following heat exposure across all treatments, although the magnitude of this difference varied among diets (*F*(4, 10) = 19.69, FDR-adjusted *p* = 1.37 × 10^−4^).

Figure S2 summarizes the direction and magnitude of these heat-associated differences. Overall, the results demonstrate diet-dependent transcriptional regulation across multiple metabolic, immune, oxidative-defense, and cellular stress-response pathways.

### 3.5. Diet-Dependent Differences in Immune-Derived Predicted Biological Age

**Figure 7:**
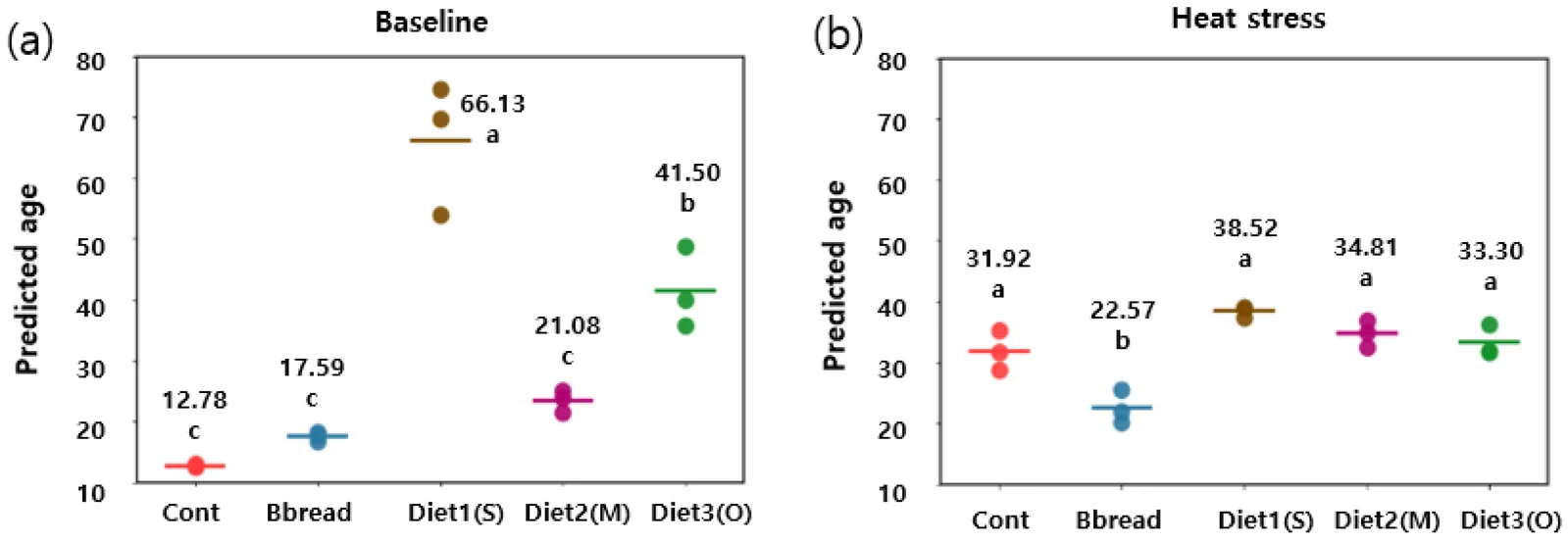
Immune-derived predicted biological age of honey bees under baseline and acute heat-stress conditions. (**a**) Predicted biological age after five days of feeding under baseline conditions and (**b**) following acute heat exposure at 45 °C for 4 h. Values represent model-derived estimates expressed in days rather than chronological age. Dots represent biological replicates (*n* = 3 cages per diet), and horizontal lines indicate group means. Different lowercase letters indicate significant differences among diets within each condition (Tukey’s HSD, *p* < 0.05).

Immune-derived predicted biological age differed significantly among diets under both baseline and post-heat conditions (Figure 7). At baseline, Diet1(S) had the highest value (66.13 ± 10.72 days), followed by Diet3(O) (41.50 ± 6.63 days). Cont (12.78 ± 0.25 days), Bbread (17.59 ± 0.74 days), and Diet2(M) (21.08 ± 0.60 days) had lower values and did not differ from one another (Figure 7a). Thus, Diet2(M) had the lowest predicted value among the pollen substitute diets at baseline.

Following acute heat exposure, separation among diets was less pronounced (Figure 7b). Bbread showed the lowest predicted biological age (22.57 ± 2.78 days) and differed significantly from all other dietary treatments. Cont (31.92 ± 3.24 days), Diet1(S) (38.52 ± 0.99 days), Diet2(M) (34.81 ± 2.25 days), and Diet3(O) (33.30 ± 2.62 days) did not differ significantly from one another.

### 3.6. Effects of Dietary Treatments on Honey Bee Survival

**Figure 8:**
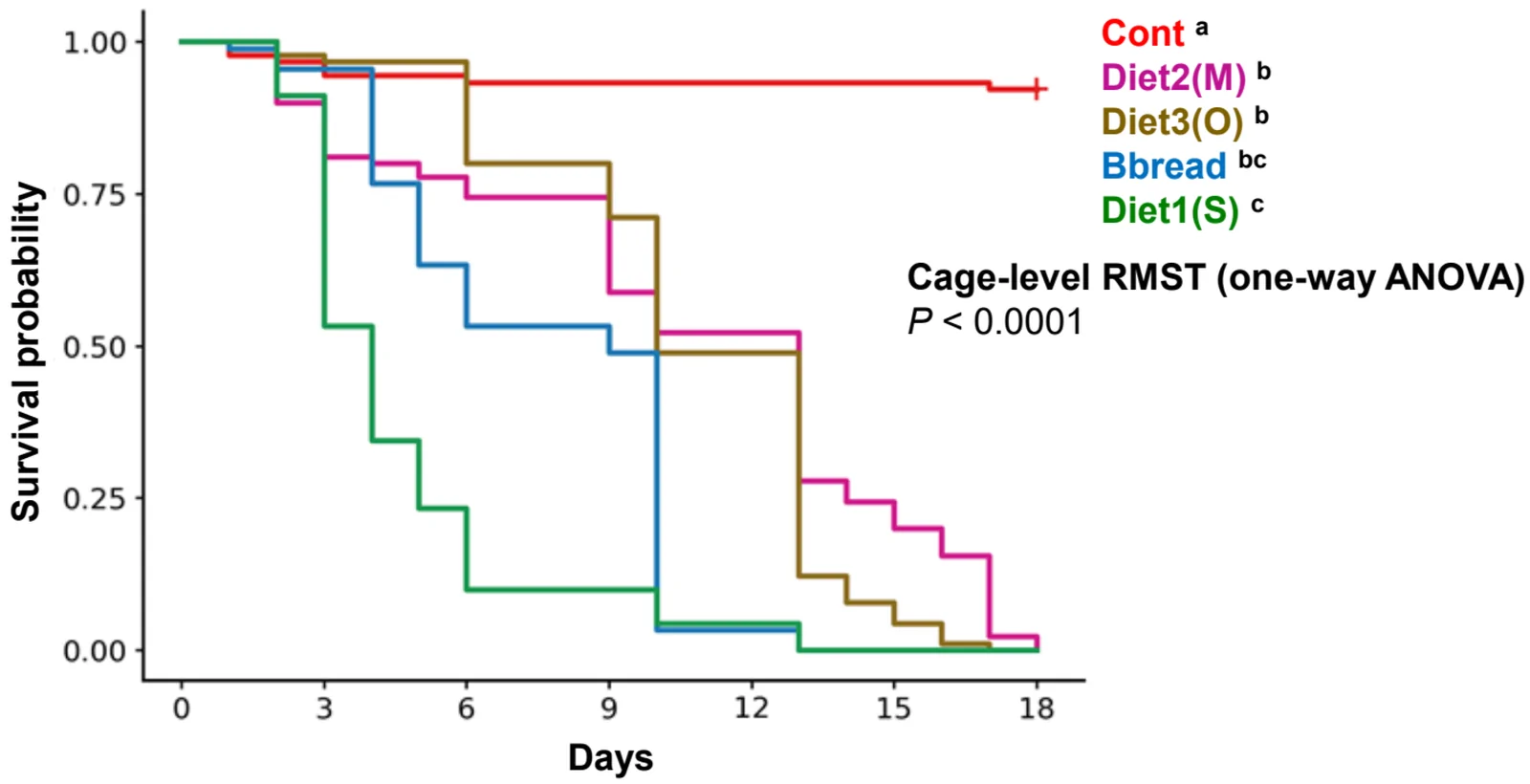
Kaplan–Meier survival curves of honey bees assigned to different dietary treatments. Survival was monitored for 18 days under laboratory conditions without heat exposure (three cages per treatment; 30 workers per cage). Kaplan–Meier curves show individual-level survival observations pooled by treatment, whereas statistical comparisons were based on restricted mean survival time (RMST) truncated at day 18 and calculated separately for each cage. Each cage served as an independent biological replicate. Cage-level RMST values were analyzed using one-way ANOVA followed by Tukey’s HSD test. Treatments not sharing a lowercase letter differed significantly (*p* < 0.05). The plus sign (+) indicates a right-censored observation.

Cage-level RMST through day 18 differed significantly among dietary treatments (*F*(4, 10) = 25.74, *p* < 0.0001; Figure 8). Cont had the highest mean RMST (16.97 ± 0.45 days; group a) and differed significantly from all other treatments. Diet2(M) (10.58 ± 1.52 days) and Diet3(O) (10.71 ± 1.89 days) did not differ significantly from each other (group b), and both had significantly higher RMST values than Diet1(S) (4.56 ± 2.24 days; group c). Bbread had an intermediate RMST (7.49 ± 1.15 days; group bc) and did not differ significantly from Diet1(S), Diet2(M), or Diet3(O). Thus, the cage-level analysis indicated higher RMST values for Diet2(M) and Diet3(O) than for Diet1(S), whereas Bbread was statistically indistinguishable from all three pollen substitute treatments. Values are presented as mean ± SD.

## 4. Discussion

Diet-dependent differences were observed in transcriptional profiles, immune-derived predicted biological age under both baseline and post-heat conditions, as well as in survival measured in an independent feeding assay conducted without heat exposure. Although apparent consumption did not differ among the three pollen substitutes, their transcriptional profiles, predicted biological age estimates, and survival outcomes varied among formulations. These findings demonstrate the value of integrating apparent consumption, transcriptional responses, immune-derived predicted biological age, and survival outcomes when evaluating pollen substitute diets.

### 4.1. Diet2(M) Exhibits a Favorable Baseline and Survival Profile Without Greater Apparent Consumption

Among the pollen substitutes, Diet2(M) showed a favorable combination of baseline molecular indicators and survival performance in the independent non-heat assay, despite having the numerically lowest apparent consumption. Apparent diet consumption, estimated from diet disappearance, may be influenced by diet palatability and physical properties and does not directly quantify actual nutrient intake, digestibility, or post-ingestive nutrient utilization [8,22,29]. Because dry-matter and nutrient intake were not quantified, the nutritional basis of this pattern remains uncertain. Nevertheless, the absence of greater apparent consumption indicates that the molecular and survival outcomes of Diet2(M) cannot be explained simply by greater apparent diet consumption.

At baseline, Diet2(M) clustered with Cont and occupied a similar region of the multivariate space associated with positive loadings for nutrient-responsive genes, including *ilp1*, *ilp2*, and *tor*. In contrast, Diet1(S) and Diet3(O) clustered together and were positioned toward genes involved in immune and oxidative-defense responses, including *relish*, *apid1*, and *sod1*. These patterns indicate that the diets established different baseline transcriptional profiles before heat exposure. However, higher expression of individual immune or oxidative-defense genes does not necessarily indicate a beneficial physiological state, just as lower expression does not necessarily indicate an adverse state. Baseline expression may instead reflect differences in pathway activation, compensatory regulation, or responses to underlying nutritional and physiological conditions [15,16,17,25,30].

The development of Diet2(M) was inspired by the natural microbial and biochemical processing associated with the transformation of collected pollen into bee bread during storage in honeycomb cells [9]. Based on this concept, defatted soybean powder was fermented with *Bacillus subtilis* MBLB2796 to explore whether microbial processing could improve its suitability as a protein source for honey bees. Fermentation can alter soybean nutrient availability and bioactivity [31]. However, Diet1(S) also contained a *Bacillus*-fermented soybean-derived ingredient, yet the two diets produced different molecular and survival profiles. Therefore, the observed performance of Diet2(M) cannot be attributed to fermentation alone; differences in the starting substrate, microbial strain, fermentation conditions, processing methods, and resulting ingredient composition may also have contributed. Previous work associated Diet2(M) with relatively high digestibility, prolonged survival, and a distinct gut microbial profile [32], whereas MBLB2796 exhibited hydrolytic, antimicrobial, and antioxidant activities in vitro [26]. These findings support further evaluation of the nutritional and functional properties of Diet2(M) but do not establish probiotic activity in honey bees because bacterial viability in the diet and persistence in the gut were not evaluated.

The position of Diet2(M) on the positive side of baseline F1, which was characterized by positive loadings for *ilp1*, *ilp2*, and *tor*, further links its transcriptional profile to nutrient-responsive insulin/insulin-like signaling and target of rapamycin pathways [18,19,33,34]. Its lower immune-derived predicted biological age than Diet1(S) and Diet3(O), together with the absence of significant differences from Cont and Bbread, indicates a comparatively lower model-derived immune-associated age estimate at baseline. However, this estimate represents a relative, model-derived indicator and should not be interpreted as the literal chronological or physiological age of the workers [24].

### 4.2. Dietary Context Shapes Transcriptional Responses to Acute Heat Exposure

Acute heat exposure elicited both broadly shared and diet-dependent transcriptional responses. Heat-shock proteins are conserved molecular chaperones that stabilize damaged proteins, limit protein aggregation, and facilitate protein refolding or removal, thereby supporting cellular homeostasis during thermal stress [35]. The increase in *hsp70* expression across dietary treatments was therefore consistent with activation of a conserved cellular heat-shock response. However, the significant diet × heat interaction for *hsp70* indicated that the magnitude of this response differed among diets. Thus, a shared direction of response did not imply a uniform transcriptional response across treatments.

Expression of *dome* increased after heat exposure across all dietary treatments. The *dome* encodes a receptor in the JAK–STAT pathway and has been implicated in honey bee antiviral immunity. Greater *dome* transcript abundance has been associated with lower viral loads in field colonies, although this association does not demonstrate causality [36]. Because heat exposure can alter cellular and humoral immune processes in honey bees [37], the overall increase in *dome* expression was consistent with heat-associated modulation of immune signaling. However, the diet × heat interaction for *dome* was not significant (FDR-adjusted *p* = 0.487); therefore, the present data provide no evidence that the magnitude of this response differed among diets.

More broadly, the significant diet × heat interactions detected for 10 of the 12 measured genes indicated that diet modified the magnitude and/or direction of early transcriptional responses involving metabolic, endocrine, immune, cellular stress-response, and oxidative-defense pathways. This finding is consistent with evidence that nutritional and thermal stressors engage overlapping physiological pathways in honey bees [38,39]. Diet-dependent survival under elevated temperatures has also been reported [6]; however, whether the transcriptional differences observed in the present study translate into greater thermal resilience remains to be tested directly.

The multivariate configuration observed after heat exposure differed from that at baseline. Cont and Bbread clustered together after heat exposure, whereas Diet1(S) and Diet3(O) formed a separate cluster; Diet2(M) remained separated from the beebread-based reference treatments and occupied a comparatively distinct position among the pollen substitutes. Consistent with this multivariate pattern, the hierarchical clustering heatmap showed a clear post-heat separation between the natural, beebread-based reference treatments and the three artificial pollen substitute diets (Figure 4b). This concordance between the multivariate ordination and heatmap analyses suggests that acute heat exposure revealed or accentuated broad differences in transcriptional organization between the natural and artificial dietary groups. These differences may reflect variation in secondary metabolites, micronutrients, or other pollen- and beebread-derived bioactive components that contribute to molecular stress regulation [40,41]. This interpretation remains hypothetical, however, because these components were not measured directly, and the observed clustering does not identify the factors responsible for the separation.

The comparatively smaller increase in *hsp70* expression in Diet2(M)-fed honey bees likewise cannot be classified as favorable or unfavorable. It could reflect lower proteotoxic stress, reduced cellular responsiveness, differences in baseline expression, or variation in the timing of transcriptional activation [21,42]. Moreover, no mortality occurred during the 4 h heat exposure, and post-exposure survival and recovery were not assessed. Therefore, the observed transcriptional differences demonstrate diet-dependent early molecular responses to acute heat exposure but do not establish that any diet conferred superior heat tolerance.

### 4.3. Immune-Derived Predicted Biological Age and Survival Provide Complementary Indicators

Immune-derived predicted biological age was estimated from the expression of *relish*, *sod1*, *apid1*, and *dome*, normalized using the reference gene *β-actin*, and integrated using the trained elastic net regression model [24]. At baseline, Diet2(M) showed a lower predicted value than Diet1(S) and Diet3(O) and did not differ significantly from Cont or Bbread. This pattern broadly agreed with the baseline multivariate analysis and indicated that the model-derived values for Diet2(M)-fed honey bees more closely resembled those of the reference treatments than those of the other pollen substitute groups. Because the model is based on age-associated immune and antioxidant gene expression, its estimates represent relative differences in immune-associated transcriptional state among treatments rather than the actual chronological or physiological ages of the honey bees [43,44].

After acute heat exposure, differences among the pollen substitute groups became less pronounced. Acute heat exposure can rapidly alter immune, antioxidant, and cellular stress-response pathways; consequently, the post-heat estimates are more appropriately interpreted as context-dependent model outputs influenced by stress-associated transcriptional changes rather than as evidence of accelerated or reversed aging [45]. The convergence of these estimates after heat exposure further indicates that the biological interpretation of the model-derived values depends on physiological context.

The independent survival assay provided a complementary functional measure of nutritional performance. Diet1(S) combined the highest baseline predicted biological age with the lowest survival, whereas Diet2(M) combined a relatively low baseline value with significantly higher survival than Diet1(S). This directional concordance suggests that the two endpoints may capture partly overlapping aspects of diet performance under the experimental conditions. However, no formal association or predictive relationship was established, and the pattern was not consistent across all diets. The two endpoints should therefore be viewed as complementary rather than interchangeable: predicted biological age reflects a selected immune-associated transcriptional state at a single sampling point, whereas survival reflects the cumulative organism-level effects of diet over time.

Because survival was evaluated in an independent assay without heat exposure, it represents general nutritional performance under cage conditions rather than heat resilience. Furthermore, the study design cannot determine whether early cage-level estimates of predicted biological age forecast subsequent survival in the corresponding populations. Longitudinal experiments linking early molecular measurements with later survival, post-heat recovery, and colony performance within the same experimental populations are needed to evaluate the functional and predictive utility of this model-derived indicator.

### 4.4. Limitations and Practical Implications

The cage experiment used newly emerged workers, included only three biological replicates per diet, and did not reproduce the social organization or colony-level thermoregulation of field colonies [46]. In addition, the relatively high incubation temperature (33 ± 2 °C) may have influenced baseline physiological and transcriptional responses; therefore, the findings should be interpreted within the specific thermal conditions of this cage experiment. Apparent consumption was estimated from diet disappearance and may have been affected by evaporation, spillage, and differences in the physical properties of the diets. Because apparent consumption was not normalized to bee-days, variation in the number of surviving workers during the feeding period may also have influenced the cage-level estimates. Direct measurements of dry-matter intake, nutrient digestibility, fermentation-derived metabolites, and pollen- and beebread-derived bioactive compounds are needed to clarify the mechanisms underlying the observed responses. In addition, the normalized gene expression results should be interpreted cautiously because *β*-*actin* was used as the sole reference gene and its stability across dietary and thermal conditions was not independently validated.

The applicability and generalizability of the immune-derived predicted biological age model require further validation across additional diets, worker age groups, stressors, and colony conditions. Furthermore, transcription was measured at only one time point after heat exposure; no mortality occurred during the 4 h exposure, and post-exposure survival and recovery were not monitored. The independent survival assay did not include heat exposure. Consequently, the functional implications of the observed molecular responses for heat tolerance remain unresolved.

Within these limitations, the combined evidence from baseline transcriptional profiles, immune-derived predicted biological age, apparent consumption, and survival under non-heat conditions supports prioritizing Diet2(M) for further evaluation. However, the present cage-based findings do not establish its efficacy under field conditions or demonstrate superior heat tolerance. Future studies should quantify nutrient intake and diet composition, examine molecular responses at multiple time points during and after heat exposure, monitor post-stress recovery and survival, and evaluate colony-level performance under field-relevant conditions. These studies will help clarify the mechanisms underlying the observed responses, assess the practical performance of Diet2(M), and determine whether immune-derived predicted biological age has functional or predictive value as an early screening indicator.

## 5. Conclusions

Pollen substitute formulation influenced the baseline transcriptional profiles of honey bees and their early molecular responses to acute heat exposure. Among the substitutes evaluated, Diet2(M) exhibited a baseline transcriptional profile similar to that of Cont, a lower immune-derived predicted biological age than Diet1(S) and Diet3(O), and a higher cage-level RMST than Diet1(S), despite comparable apparent consumption among the substitutes. Following heat exposure, the beebread-based reference treatments and pollen substitutes showed broadly different transcriptional patterns, indicating diet-dependent molecular responses. However, because no mortality occurred during heat exposure and post-exposure recovery and survival were not assessed, improved heat tolerance could not be established. Overall, these findings support prioritizing Diet2(M) for colony-level evaluation and demonstrate the potential value of integrating transcriptional profiles and immune-derived predicted biological age with consumption and survival outcomes for preliminary diet screening. The predictive utility of the biological age indicator and the capacity of candidate diets to support heat resilience require further validation under colony-level and field-relevant conditions. Given the increasing risk of extreme heat associated with climate change, developing nutritionally balanced supplemental feeds that help maintain honey bee health under elevated temperatures represents an important research priority.

## 6. Patents

A Korean patent application related to the diet formulations evaluated in this study was filed on 12 December 2025 under the title “Feed Composition for Promoting Bee Colony Growth” (Korean Patent Application No. 10-2025-0197548).

## Figures and Tables

**Table 1:** Soybean-derived ingredients used in the experimental pollen substitute diets.

Diet	Diet-Specific Soybean-Derived Ingredient	Inclusion Level (%)
Diet1(S)	*Bacillus*-fermented soybean-derived product (Soytide powder)	2
Diet2(M)	Defatted soybean powder fermented with *Bacillus subtilis* MBLB2796	2
Diet3(O)	Non-fermented defatted soybean powder	2

**Table 2:** Apparent consumption of beebread and pollen substitute diets during the five-day feeding period.

Dietary Treatment	Apparent Consumption (g/Cage/5 Days)
Bbread	1.765 ± 0.158 ^a^
Diet1(S)	1.307 ± 0.045 ^b^
Diet2(M)	1.069 ± 0.197 ^b^
Diet3(O)	1.266 ± 0.071 ^b^

Values are presented as the mean ± SD of three biological replicates per dietary treatment. Different lowercase superscript letters indicate significant differences among treatments according to Tukey’s HSD test (*p* < 0.05). Cont was excluded because beebread and liquid sugar syrup were provided in separate feeders, preventing direct comparison of total diet disappearance with the other treatments.

## Data Availability

The data presented in this study are available in the article and its Supplementary Materials.

## Supplementary Materials

The following supporting information can be downloaded at: https://www.mdpi.com/article/10.3390/ani16193147/s1. Figure S1: PCAmix representation of transcriptional profiles under baseline and acute heat-exposure conditions; Figure S2: Diet-specific transcriptional responses to acute heat exposure; Table S1: Analyzed proximate and mineral composition of the experimental pollen substitute diets; Table S2: PCR primer sequences; Table S3: Effects of diet, thermal condition, and their interaction on gene expression in honey bees.
