# Supplementary material for: Integrated Assessment of Pollen Substitute Diets in Honey Bees Based on Transcriptional Responses, Immune-Derived Predicted Biological Age, and Survival
Source: Animals (Basel). 2026 Oct 8;16(19):3147. doi: 10.3390/ani16193147 (PMC13663437; doi:10.3390/ani16193147)
Supplement: Supplementary file 1 [file animals-16-03147-s001.zip › 0915_Diet2_Suppliment.pdf]

Table S1. Analyzed proximate and mineral composition of the experimental pollen substitute diets

Component	Diet1(S) (%)	Diet2(M) (%)	Diet3(O) (%)
Moisture	32.14	32.71	31.69
Crude protein	17.77	17.76	17.77
Crude fat	4.98	4.89	4.91
Crude fiber	0.01	0.31	0.13
Crude ash	2.59	2.72	2.58
Calcium	0.14	0.15	0.15
Phosphorus	0.54	0.54	0.57

Proximate and mineral compositions were analyzed by the Center for Agricultural Science and Technology, Chungnam National University, Daejeon, Republic of Korea. Values are expressed as percentages on an as-submitted basis.

Table S2. PCR primer sequences.

No.	Gene	Primer	Sequence (5′–3′)	Amplicon length (bp)	NCBI reference sequence
1	*ilp1*	F	CGATAGTCCTGGTCGGTTTG	237	XM_026442144.1
2		R	CAAGCTGAGCATAGCTGCAC		
3	*ilp2*	F	TTCCAGAAATGGAGATGGATG	166	XM_026444890.1
4		R	TAGGAGCGCAACTCCTCTGT		
5	*tor*	F	AACAACTGTTGCTGACGGTG	153	XM_006566642.3
6		R	GTTGCAGTCCAGGCTTTTTG		
7	*jhamt*	F	TTGGACATAGGTTGCGGACC	302	NM_001327967.1
8		R	AATCCTTTTCCTCCTGGCCG		
9	*vg*	F	GCAGAATACATGGACGGTGT	146	NM_001011578.1
10		R	GAACAGTCTTCGGAAGCTTG		
11	*AmGr10*	F	TCTAGCGAACGCGACCGAAA	174	XM_006567110.3
12		R	AGGTTCCACGCGGAATCAGT		
13	*dome*	F	GCCGCTGCTCTTTGGCATCT	238	XM_006567690.3
14		R	GCCAAATTGTTGTTCCAACAGCCC		
15	*relish*	F	TCCATTGCATGCAGCACTTCG	264	XM_026444175.1

No.	Gene	Primer	Sequence (5′–3′)	Amplicon length (bp)	NCBI reference sequence
16		R	ACACATGCACCAGCTTCAGGA		
17	*defensin2*	F	ACCGCTGCTACCACTACGACA	139	NM_001011638.1
18		R	GCCATTTCTGCAACTACCGCCT		
19	*apid1*	F	TTGTTGTTACCTTTGTAGTCGCGGT	70	NM_001011642.1
20		R	AGGCGCGTAGGTCGAGTAGG		
21	*sod1*	F	GCAGTGTGCGTTCTTCAGGGT	86	NM_001178027.1
22		R	TGACCGGTGACCTTCACGGA		
23	*hsp70*	F	ATCAACCTGGCGTCTTGATTC	104	NM_001160072.2
24		R	AGGTGCAGGTGGAATGCCAG		
25	*β-actin*	F	GGATTCCTATGTTGGTGATGAAGCCC	177	NM_001185145.1
26		R	GGTGCCTCAGTAAGAAGTACCGGATG		

Table S3. Effects of diet, thermal condition, and their interaction on gene expression in honey bees.

Gene	Effect	F	*P*	df1	df2	*FDR-adjusted P*
ilp1	Diet	169.63	3.83×10^{-9}	4	10	—
ilp1	Heat stress	43.95	5.86×10^{-5}	1	10	—
ilp1	Diet × heat stress	10.22	0.00147	4	10	1.76×10^{-3}
ilp2	Diet	162.29	4.76×10^{-9}	4	10	—
ilp2	Heat stress	87.14	2.98×10^{-6}	1	10	—
ilp2	Diet × heat stress	19.50	1.03×10^{-4}	4	10	1.37×10^{-4}
tor	Diet	19.87	9.49×10^{-5}	4	10	—
tor	Heat stress	0.45	0.518	1	10	—
tor	Diet × heat stress	2.44	0.116	4	10	0.126
jhamt	Diet	24.07	4.08×10^{-5}	4	10	—
jhamt	Heat stress	6.96	0.0248	1	10	—
jhamt	Diet × heat stress	59.99	5.94×10^{-7}	4	10	3.57×10^{-6}
vg	Diet	107.68	3.54×10^{-8}	4	10	—
vg	Heat stress	0.94	0.356	1	10	—
vg	Diet × heat stress	37.12	5.69×10^{-6}	4	10	1.71×10^{-5}
AmGr10	Diet	382.55	6.88×10^{-11}	4	10	—
AmGr10	Heat stress	12.45	0.00546	1	10	—

Gene	Effect	F	*P*	df1	df2	*FDR-adjusted P*
AmGr10	Diet × heat stress	37.95	5.13×10^{-6}	4	10	1.71×10^{-5}
relish	Diet	59.52	6.17×10^{-7}	4	10	—
relish	Heat stress	0.82	0.386	1	10	—
relish	Diet × heat stress	22.94	5.05×10^{-5}	4	10	1.02×10^{-4}
dome	Diet	12.86	5.93×10^{-4}	4	10	—
dome	Heat stress	6.16	0.0325	1	10	—
dome	Diet × heat stress	0.93	0.487	4	10	0.487
defensin2	Diet	64.82	4.11×10^{-7}	4	10	—
defensin2	Heat stress	5.76	0.0373	1	10	—
defensin2	Diet × heat stress	22.10	5.96×10^{-5}	4	10	1.02×10^{-4}
apid1	Diet	106.52	3.73×10^{-8}	4	10	—
apid1	Heat stress	57.72	1.85×10^{-5}	1	10	—
apid1	Diet × heat stress	96.11	6.15×10^{-8}	4	10	7.38×10^{-7}
sod1	Diet	66.93	3.52×10^{-7}	4	10	—
sod1	Heat stress	0.39	0.544	1	10	—
sod1	Diet × heat stress	22.10	5.96×10^{-5}	4	10	1.02×10^{-4}
hsp70	Diet	46.87	1.91×10^{-6}	4	10	—
hsp70	Heat stress	1807.72	1.24×10^{-12}	1	10	—
hsp70	Diet × heat stress	19.69	9.88×10^{-5}	4	10	1.37×10^{-4}

Mixed-design repeated-measures ANOVA was applied to ΔCt values, with diet as the between-cage factor, thermal condition as the within-cage factor, and cage nested within diet as the repeated-measures subject. FDR-adjusted *P* values were calculated across the 12 genes for the diet × thermal condition interaction using the Benjamini–Hochberg procedure. An em dash indicates that FDR correction was not applied to the corresponding main effect.

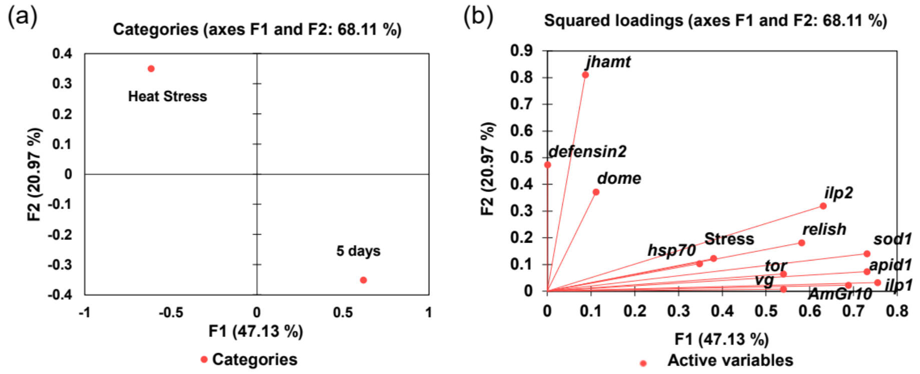

(c)

Gene	F1	F2
ilp1	0.756	0.032
ilp2	0.631	0.320
tor	0.540	0.065
jhamt	0.087	0.810
vg	0.540	0.008
AmGr10	0.689	0.023
relish	0.581	0.182
dome	0.112	0.372
defensin2	0.001	0.474
apid1	0.731	0.074
sod1	0.731	0.140
hsp70	0.348	0.104
Stress	0.380	0.123

Figure S1. PCAmix representation of transcriptional profiles under baseline and acute heat-exposure conditions. (a) Projection of the thermal-condition categories onto the first two dimensions, illustrating the separation between baseline and post-heat conditions. (b) Squared-loading plot showing the contributions of the 12 gene-expression variables and thermal condition to the first two PCAmix dimensions. (c) Squared-loading values of the individual variables for F1 and F2. Squared-loading values ≥0.36, corresponding to an absolute loading magnitude of ≥0.60, are highlighted in red and were considered salient contributors to each dimension. F1 (47.13%) and F2 (20.97%) collectively explained 68.11% of the total variation.

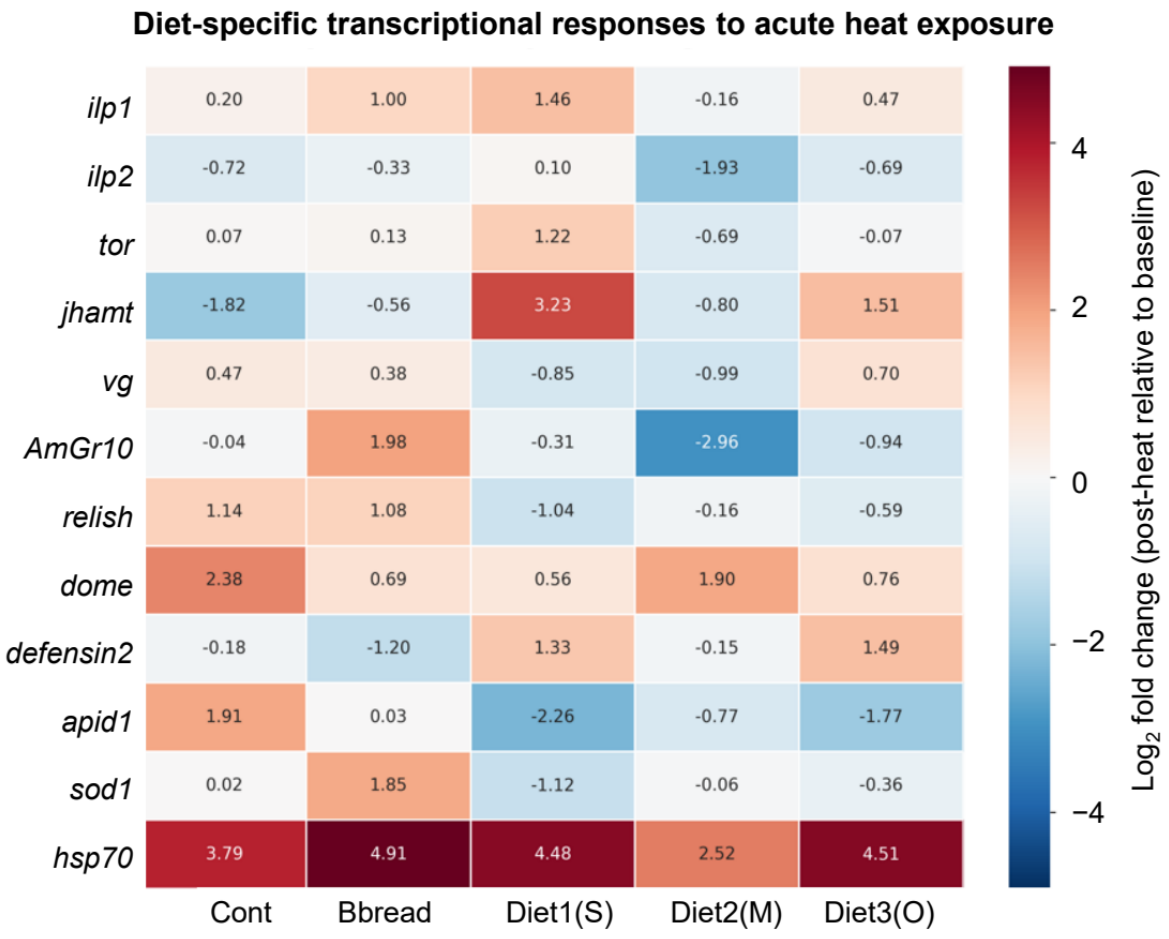

Figure S2. Diet-specific transcriptional responses to acute heat exposure. Values represent the mean $\log_2$ fold change in gene expression following acute heat exposure relative to the corresponding baseline condition for each dietary treatment (n = 3 cages per treatment). Positive values (red) indicate increased expression after heat exposure, whereas negative values (blue) indicate decreased expression. Statistical significance was evaluated using the diet × heat interaction analysis reported in Table S3.
